# ﻿*Indigoferavallicola* (Fabaceae), a new species from Yunnan, southwest China

**DOI:** 10.3897/phytokeys.199.85437

**Published:** 2022-06-03

**Authors:** Jin-Li Liu, Shi-Gang Li, Feng Yang, Huan-Chong Wang

**Affiliations:** 1 School of Life Sciences, Yunnan University, Kunming 650091, China; 2 School of Ecology and Environmental Science, Yunnan University, Kunming 650091, China; 3 Herbarium of Yunnan University, Kunming 650091, Yunnan, China

**Keywords:** Dry-hot valley, endemism, *Indigoferarigioclada*, Leguminosae, prostrate shrub

## Abstract

*Indigoferavallicola* (Fabaceae), a new species is described and illustrated. This plant is only found from two localities in the central Yunnan Province, southwest China. It is characterized by having the prostrate habit, usually 13–17-foliolate leaves and the relatively small (3–5 mm long) flowers. Morphological comparisons with its closest relatives, *I.rigioclada*, *I.franchetii*, *I.chaetodonta*, and *I.henryi* are also presented.

## ﻿Introduction

The genus *Indigofera* L., comprising approximately 750 species, is the third largest genus after *Astragalus* and *Acacia* s.l. in the legume family (Fabaceae) (Schrire et al. 2005, 2009), and composes one of the 50 largest genera of angiosperms (Frodin 2004). Species of *Indigofera* are mostly shrubs, except some are small trees or herbaceous perennials or annuals. It has a near worldwide distribution; nevertheless centers of species diversity primarily occur in Africa and Madagascar (ca. 550 species), Asia, especially the temperate Sino-Himalayan region (ca. 105 species), Australia (ca. 50 species), and the New World (ca. 45 species) (Schrire et al. 2009).

China possesses a rich set of species of *Indigofera*, and the highest species diversity was found in the southwest region (Yin et al. 1992). One hundred years ago, Craib (1913) made the first comprehensive revision of Chinese *Indigofera*. In his treatment, 57 species were recognized from China, 31 species of which were newly named. In the most recent revision by Gao and Schrire (2010) for the “Flora of China”, 79 species and 9 varieties have been recognized, including 45 endemics. More recently, two additional new species of *Indigofera* were described from southwest China by Zhao and Gao (2015) and Zhao et al. (2020) respectively; these findings highlight the need for continued field exploration and taxonomical research in this area.

During recent field surveys in Yunnan Province (SW China), we came across an unknown species of *Indigofera* in the Luzhijiang valley. After detailed comparison with its morphologically similar species, it became clear that this plant represents a distinct new species.

## ﻿Materials and methods

The study followed the normal practice of plant taxonomic survey and herbarium taxonomy. Morphological studies of the new species were based on observation of living plants and specimens housed at PYU and YUKU. Digital images of type specimens of the genus *Indigofera* available at JSTOR Global Plants (http://plants.jstor.org/), as well as collections housed at CDBI, KUN, PE, PYU and YUKU, were extensively examined and compared with the new species. Pertinent taxonomic literature (e.g. Fang and Zheng 1994; Sun 2006; Gao and Schrire 2009, 2010; Chauhan et al. 2013; Clark et al. 2015) were extensively consulted. Measurements were carried out under a stereomicroscope (Olympus SZX2, Tokyo, Japan) using a ruler and a metric vernier caliper.

## ﻿Taxonomy

### ﻿ Indigofera vallicola

Taxon classificationPlantaeFabalesFabaceae

Huan C. Wang & Jin L. Liu sp. nov.

B9021464-0B8C-5D87-AD0E-4F47457146D2

urn:lsid:ipni.org:names:77299060-1

Figs 1 , 2 , 3 , 4

#### Type.

China. Yunnan Province: Yimen County, Luzhi Town, Luzhijiang valley, Xiao Luzhi, 24°24'N, 101°34'E, alt. 1,320 m, 25 September 2021, *Huan-Chong Wang et al. YM15303* (Holotype: YUKU!; isotypes: YUKU!)

#### Diagnosis.

*I.vallicola* is most similar to *I.rigioclada* Craib by sharing the procumbent habit, relatively small leaves and the similar flower shape, but it clearly differs from the latter by its usually 13–17-foliolate, flowers 3–5 mm long, calyx teeth triangular-lanceolate, and legumes 1–2 mm in diameter.

**Figure 1.**
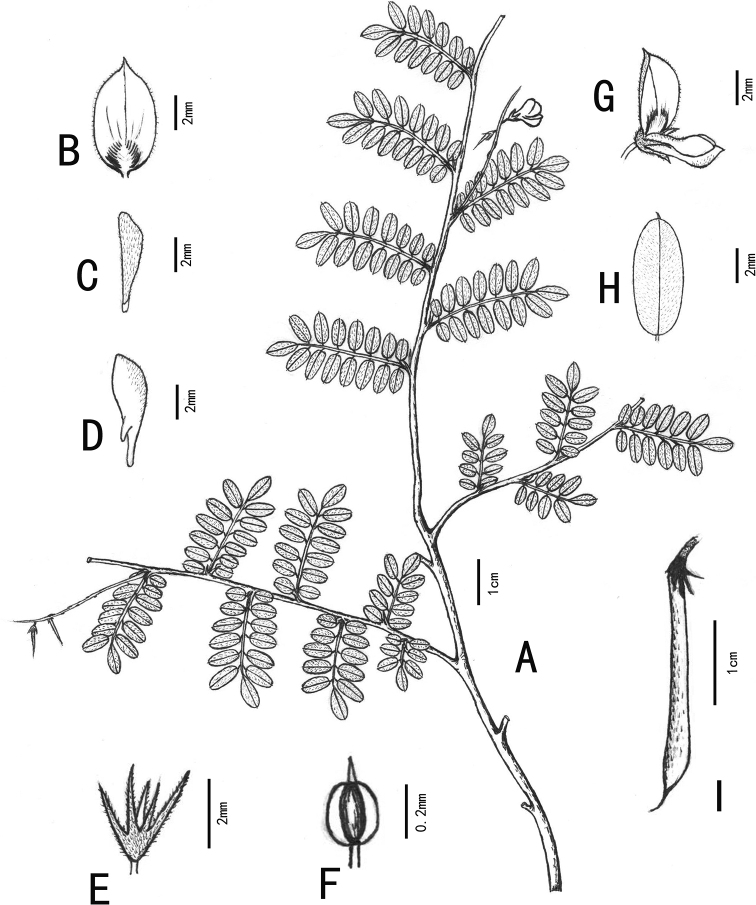
*Indigoferavallicola* Huan C. Wang & Jin L. Liu sp. nov. (Drawn by Jin-Li Liu) **A** habit **B** standard **C** wing **D** keel **E** calyx **F** anther **G** flower **H** leaflet **I** legume.

#### Description.

Dwarf shrubs, usually prostrate, 20–35 cm in height. Stems branched, brown, glabrescent, usually 1–2.5 mm in diameter. Branchlets nearly terete, 10–15 cm long, with dense appressed white and brown medifixed and symmetrically 2-branched trichomes. Leaves imparipinnate, 2–5 cm long, 1–2 cm wide, usually (7–)13–17-foliolate. Stipules lanceolate to subulate, 1–2 mm long. Petioles 0.2–0.4 cm long, petioles and rachis subterete, adaxially grooved, with appressed white and brown medifixed symmetrically 2-branched trichomes. Leaflets opposite, 0.2–1.2 cm long, 0.15–0.5 cm wide, adaxially green, abaxially gray, midvein abaxially prominent and adaxially impressed, secondary veins inconspicuous, both surfaces with white and brown medifixed symmetrically 2-branched trichomes; terminal leaflets obovate, apex rounded to truncate, and mucronate, base cuneate; lateral leaflets oblong or elliptic, apex rounded to truncate and mucronate, base rounded. Inflorescences racemose, axillary, 2.5–6 cm long. Peduncles 1–1.8 cm long. Bracts caducous, lanceolate to ovate-lanceolate, purple, ca. 0.2 cm long, abaxially with white medifixed trichomes, adaxially glabrous. Pedicels 1–2 mm long. Calyx funnelform, rarely cup-shaped, purple, outside with white and brown medifixed symmetrically 2-branched trichomes, glabrous inside; tube ca. 1 mm long; teeth 5, unequal, triangular-lanceolate, ca. 1 mm long, apex acuminate. Corolla pink; standard obovate, 3–5 mm long, 2–3 mm wide, apex mucronate, outside with white medifixed trichomes; wings spoon-shaped, 2.5–4.0 mm long, ca. 1 mm wide, outside pilose; keels 3–5 mm long, ca. 1 mm wide, outside pilose, with a small lateral spur. Stamens 3–5 mm long, anthers broadly ovoid, apex mucronate. Ovary hairy, style glabrous. Legumes linear, cylindric, 1.5–3.2 cm long, 0.1–0.2 cm in diameter, apex beaked, with white and brown medifixed symmetrically 2-branched trichomes. Seeds usually 6–8, oblong to rectangle, dark-brown, glabrous, 1–2 mm long, ca. 1 mm wide.

**Figure 2.**
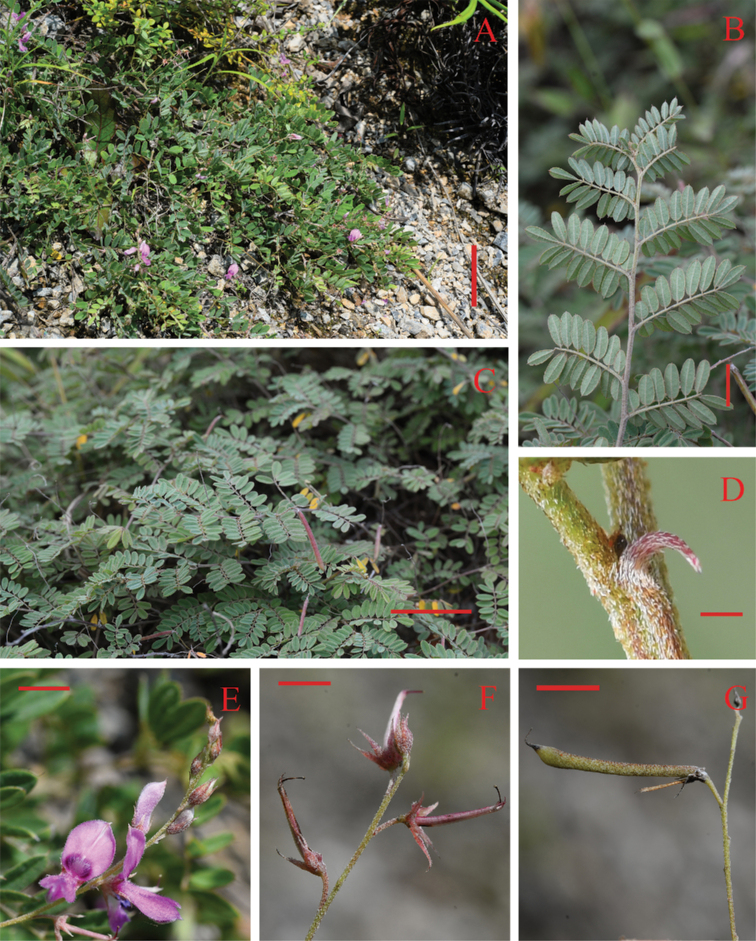
*Indigoferavallicola* Huan C. Wang & Jin L. Liu sp. nov. **A** habit **B** a portion of branchlet showing abaxial surfaces of leaflets **C** plants in fruiting stage **D** stipules **E** a portion of inflorescens **F** calyces **G** legume. Scale bars: 10 cm (**A**); 4 cm (**C**); 1 cm (**B, G**); 4 mm (**E**); 3 mm (**F**); 1 mm (**D**).

#### Phenology.

Flowering occurs from September to November, fruiting from October to December.

#### Distribution and ecology.

*Indigoferavallicola* is endemic to southwest China, where it has only been collected from two localities (ca. 45 km apart from each other) in central Yunnan to date: Xiao Luzhi (type locality) in Luzhijiang valley and Ainishan village in Shuangbai County. The climate in its habitat is seasonally hot and arid. In the type locality, *I.vallicola* occurs in the xerophilous scrubs or grasslands at elevations of 1200–1800 m, and its association include *Phyllanthusemblica* Linn. (Phyllanthaceae), *Paliurusorientalis* (Franch.) Hemsl. (Rhamnaceae), *Dalbergiayunnanensis* Franch. (Fabaceae), *Symphoricarpossinensis* Rehd. (Caprifoliaceae), *Duhaldealachnocephala* Huan C. Wang & Feng Yang (Asteraceae) (an endemic species described by Yang et al. (2022)), *Pterygiellaluzhijiangensis* Huan C. Wang (Orobanchaceae), *Sileneotodonta* Franch. (Caryophyllaceae), *Spodiopogonsagittifolius* Rendle (Poaceae), *Heteropogoncontortus* (Linn.) Beauv. ex Roem. & Schult. (Poaceae) and *Themedacaudata* (Nees ex Hooker & Arnott) A. Camus (Poaceae).

**Figure 3.**
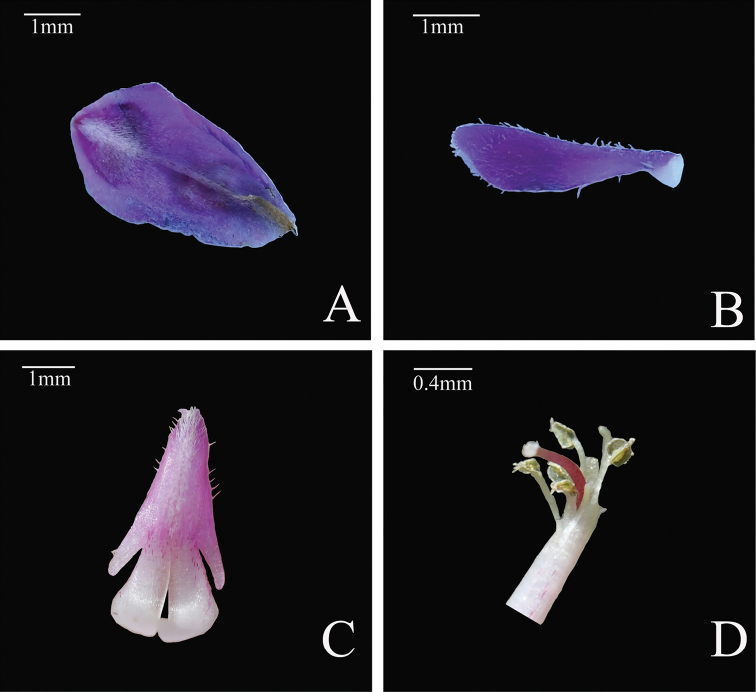
*Indigoferavallicola* Huan C. Wang & Jin L. Liu sp. nov. **A** standard **B** wing **C** keel **D** stamens and pistil.

#### Etymology.

The specific epithet is taken from the Latin “*vallis*” (valley) and the suffix “-*cola*” (dweller), referring to the habitat where the new species is found.

**Figure 4.**
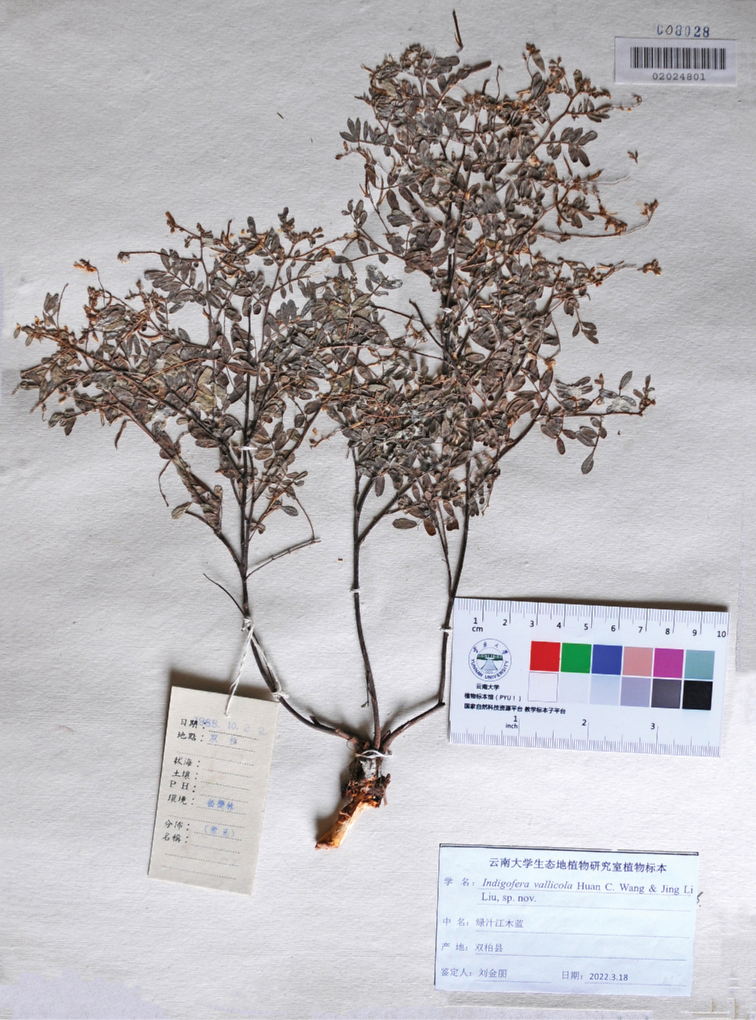
A specimen (YUKU-02024801) of *Indigoferavallicola* Huan C. Wang & Jin L. Liu sp. nov. collected in October 1965 from Ainishan village in Shuangbai County, southwest China.

#### Additional specimens examined.

**China. Yunnan**: Shuangbai County, Ainishan, alt. 1,800 m, 22 October 1965, *W. M. Zhu et al. 04195* (YUKU); Yimen County, Luzhi Town, alt. 1,250 m, 20 October 1965, *W. M. Zhu et al. 4659* (YUKU); *ibid.*, 3 October 2016, *H. C. Wang et al. YM1274* (YUKU); *ibid.*, 12 November 2019, *H. C. Wang et al. YM8322* (YUKU).

#### Taxonomic notes.

*Indigoferavallicola* is mainly characterized by having the prostrate habit, usually 13–17-foliolate leaves and relatively small (3–5 mm long) flowers. Morphologically, it is most similar to *I.rigioclada* Craib by sharing the procumbent habit, relatively small leaves and similar flower shape, but it clearly differs from the latter by its usually 13–17-foliolate (vs. 5–13-foliolate in *I.rigioclada*), flowers 3–5 mm (vs. 8–10 mm) long, calyx teeth triangular-lanceolate (vs. triangular), and legumes 1–2 mm (vs. larger than 2 mm) in diameter. *Indigoferavallicola* is also more or less similar to *I.henryi* Franch. in its overall appearance, relatively gracile pedicels and shape of calyx. Nevertheless, *I.henryi* clearly differs from the former in having the linear stipules usually 5 mm long (vs. lanceolate to subulate, 1–2 mm long in *I.vallicola*), leaves larger, 3–10 cm (vs. 2–5 cm) long, rachis of adaxially flattened, slightly winged (vs. grooved and without winged), leaflet blades 1.7–2.3 × 0.5–1.2 cm (vs. 0.2–1.2 × 0.15–0.5 cm), pedicels (2)3–6(–9) mm (vs. 1–2 mm) long, corolla much larger in size, white (vs. pink), with 7–9 × 5–6 mm (vs. 3–5 × 2–3 mm) standard and 7–9 mm (vs. 3–5 mm) long keels.

*Indigoferavallicola* is somewhat close to *I.franchetii* X. F. Gao & Schrire, an endemic species found from the dry-hot valleys of Jinsha River and its tributaries in southwestern China. Nevertheless, *I.franchetii* differs from *I.vallicola* in having 35–50 cm long branchlets, (11–)17–27-foliolate leaves ca. 5–10 cm long, racemes 5.5–11 cm long, peduncles 0.7–3.0 cm long, pedicels 0.5–1.0 mm long, calyces cup-shaped, standards oblong-elliptic, 7–8 mm long, legumes 2.5–4.0 cm long, 1.5–2.5 mm in diameter. The new species shows some similarities with *I.chaetodonta* Franch. in the habit, flower size, and calyx shape. However, *I.chaetodonta* is well differentiated from *I.vallicola* in having (5 or) 7- or 9-foliolate leaves 0.6–1.5 (–2.0) cm long, leaflet blades oblong to oblanceolate, 3.5–6 × 1.5–2.5 mm, adaxially nearly glabrous, standards broadly elliptic, 5–7 × ca. 4 mm, legumes 1.5–2.0 cm long, glabrous or with sparse appressed medifixed trichomes.

## Supplementary Material

XML Treatment for Indigofera vallicola
